# Organic Amendments: Direct Application and Residual Effects on Vegetative and Reproductive Growth of Hot Pepper

**DOI:** 10.1155/2022/2805004

**Published:** 2022-08-24

**Authors:** Dargie Tsegay Berhe, Yohannes Zergaw, Temesgen Kebede

**Affiliations:** Dilla University, College of Agriculture and Natural Resources, Dilla, Ethiopia

## Abstract

The high production potential of coffee and animals in the Gedeo zone that could produce huge amounts of coffee pulp and animal manure wastes has been polluting the environment. In this sense, this study was aimed at averting pollution and managing plant and animal wastes, focusing on the effect of coffee pulp and animal manure in the form of vermicompost, biochar, and ordinary compost on hot pepper vegetative and reproductive growth. A 15 ton per hectare of each treatment was applied in a randomized complete block design replicated three times. Vegetative and reproductive growth parameters (plant height, leaf number, number of branches, days to 50% flowering, total fresh biomass, number of fruits per plant, single fruit length, and fruit yield per hectare) were recorded, and the statistical difference was determined at 5% significance level using R-program. The result showed that there was a significant difference among treatments. Coffee pulp vermicompost prepared using *Eisenia fetida* earthworm had significantly (*P* < 0.05) higher results in plant height, leaf number, number of branches, total fresh biomass, number of fruits per plant, single fruit length, and total yield, while the minimum values were recorded in topsoil (control treatment) and animal manure compost. The direct and residual effects of vermicompost and biochar organic amendments were the potential organic fertilizers for hot pepper fast growth and to produce enormous yield, which might be due to their nature in improving soil physicochemical and biological properties as well as nutrient uptake.

## 1. Introduction

Hot pepper is the world's second most important vegetable ranking after tomatoes, which belongs to the Solanaceae family [1]. It is the most produced type of spice flavouring and color to food while providing essential vitamins and minerals. Its pungent nature is effective as a natural pest control product [2].

Peppers are grown extensively under various environmental and climatic conditions. It requires an annual crop that grows at an altitude ranging from 1400 up to 2100 m.a.s.l. Growing pepper requires soil that is well drained and rich in organic matter, as well as 600–650 mm rainfall [3]. In different parts of Ethiopia, there is pepper production for its fruit consumption in different forms as vegetables, spices, or condiments to meet daily diets [4]. Small-scale farmers produce the largest proportion of hot pepper in Ethiopia. In many areas, pepper is grown predominantly as monocrop and rotated with cereals or legumes, using the main rainy season, or in some specific rift valley areas using irrigation supplies during the dry season [5].

Variety selection and nutrient management for the production of pepper are vital to increase producers' income and boost livelihood [6]. Getahun and Habtie [4] and Chimdessa et al. [1] indicated that poor soil fertility and low-quality seed are the main reasons for poor vegetative growth performance and yield of hot pepper. Zayed et al. [7] emphasized that organic fertilizers, such as decayed leaves, compost, sawdust, or animal manure, are very important for the overall hot pepper plant development. Several researchers [8–13] reported that utilizing vermicompost improves hot pepper growth and enrich soil amendment compared to conventional fertilizer by providing highly nutritive organic fertilizer and more powerful growth promoters.

Yvonne et al. [2] and Wubalem [3] explored that pepper production is highly associated with well-drained and fertile soil, which can be improved by adding rotten farm yard or compost manures. In Ethiopia, poor crop productivity is mainly associated with poor soil fertility and loss of macronutrients via soil erosion and other natural and anthropogenic factors in different parts of southern nations [10]. Particularly in lowland areas of the Gedeo zone, there is a depleted soil fertility status derived from different natural and anthropogenic factors [14, 15].

An integrated process for organic fertilizer production has been developed to valorize the pineapple leaf [16] and lemon [17] wastes obtained from agriculture and industrial sectors. According to Kundu et al. [17], the yield of the mung seed was increased by 2.02 folds when inorganic fertilizer and biomanure were combined. The physical and chemical characteristics of the soil could also be maintained by applying biomanure to crop fields [18]. Due to the rapid pace of urbanization and industrial growth, trace metal contamination has increased dramatically in recent decades, harming the environment and ecosystem significantly [19, 20]. It is essential to find environmentally friendly, sustainable, and effective technologies to support the upcoming large-scale soil fertility action worldwide [21, 22]. It includes organic, physical, or biological to systematically clean up polluted soil, enrich barren soil, or lessen or regulate the environmental risks posed by pollutants in the soil [23]. Amending the soil with organic fertilizers, which is performed by adding vermicompost, biochar, animal manure, and other organic materials, is one of the main ways for improving the fertility status of the soil [24]. As an illustration, the use of biochar in deficient soil greatly increased fertility and promoted plant growth [25–27].

In the agricultural systems of Gedeo, there is a large amount of plant and animal products followed by accelerated expansion of coffee-processing enterprises that produce large amounts of coffee husk and environmental pollution. In the study area, there is very limited information on using organic fertilizers of vermicompost, biochar, and animal manure in combination with hot pepper vegetative growth and yielding potential. Therefore, this study aimed to explore the responses of hot pepper growth and yield parameters to the direct and residual coapplication of organic amendments.

## 2. Materials and Method

### 2.1. Study Area Description

**Figure 1:**
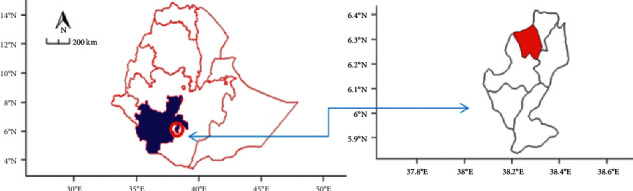
Research site (blue color = south region; red color = Wonago district).

The experiment was carried out in the Wonago district, Gedeo Zone, Southern Ethiopia, geographically located at 6^0^ 19′ 05″ north latitude and 38^0^ 15′ 36″ east longitude with 1754 m altitude and found at 376 km south of Addis Ababa (Figure 1). The district is characterized by 1001–1800 mm annual rainfall and 12–25°C temperature. The study area has suitable agroecology for vegetable, fruits, and other horticultural crops, particularly for coffee and enset. There are around twenty coffee-processing industries in the area engaged in wet and dry processing that could produce tremendous coffee pulp wastes. There was also immense animal manure waste available due to potential animal production in the area.

### 2.2. Experimental Materials and Sample Preparation

Coffee pulp and animal manure were collected from coffee-processing industries and animal farm areas, respectively. Plastics, stones, feathers, hair, bedding, and other unwanted materials were removed from the collected sample. Coffee pulp compost was prepared using 70% coffee pulp, 20% animal manure, and 10% forest soil to introduce beneficial microorganisms for decomposition as described by Solomon [28]. The prepared compost was air dried under shade, crushed, blended into powder, and screened through a 2 mm sieve and applied to the experiment. Besides, coffee pulp and animal manure biochars were produced through a slow pyrolysis process using a conventional drum, under limited oxygen conditions. Each sundried coffee pulp and animal manure was entered into drum at 560°C for 4 hours according to Ebrahimi et al. [29]. Then, the char materials were ground using mortar and pestle and sieved through a 0.5 mm sieve. Finally, the two vermicompost types were prepared from coffee pulp and animal manure (cow dung) using adult earthworms of *Eisenia fetida* and *Dendrobaena veneta* within 60 days.

### 2.3. Experimental Design and Treatments

The experiment was conducted in a randomized complete block design by directly applying 15 tons per hectare of organic amendments of coffee pulp compost (CPC), coffee pulp biochar (CPB), animal manure compost (AMC), animal manure biochar (AMB), coffee pulp fetida vermicompost (CFV), coffee pulp *Dendrobaena veneta* vermicompost (CDV), a combination of coffee pulp compost and animal manure compost (CPC_AMC), combination of coffee pulp biochar and animal manure biochar (CPB_AMB), a combination of coffee pulp fetida vermicompost and coffee pulp *Dendrobaena veneta* vermicompost (CFV_CDV) in comparison with top soil as a control treatment and replicated three times. The residual effects of the organic amendments were also evaluated as the second factor in this study.

### 2.4. Experimental Field Preparation

The experimental field was ploughed and properly leveled to have well-organized and efficient supply channels of irrigation water. The prepared organic fertilizers were applied in 20 cm depth before transplantation of hot pepper seedlings. The seedlings with four pairs of true leaves were transplanted to the well-prepared experimental plots at a spacing of 30 cm and 60 cm between plants and rows, respectively. Proper irrigation, weeding, and other good agronomic practices were applied.

### 2.5. Data Collection Procedures

Data were randomly collected from each replication to determine the growth and yield parameters of hot pepper. The number of days until 50% flower emergence was registered. The plant height was measured from ground level to the tip of the plant at the fruit matured stage using the meter and single fruit length was measured by a ruler. The average numbers of branches leaves and fruits per plant were counted in each treatment. Fresh biomass weight was recorded using a digital balance. The yield was also calculated using the following equation:

(1) $$\begin{array}{c} \text{Yield}\left(\text{tones per ha}\right)=\frac{\text{subplot yield}\left(\text{ton}\right)\times 10000\;{\text{m}}^{2}}{\left(\text{subplot area}\left({\text{m}}^{2}\right)\times 1000\right)}. \end{array}$$

### 2.6. Soil Analysis

The percentage of organic carbon (OC) was determined by the Walkley–Black dichromate method according to Bashour and Sayegh [30]. The soil pH of organic amendments was measured on a 1 : 10 (w/v) water suspension of the samples as described by Tan [31]. Following the method of Hesse [32], the total nitrogen content was determined by the micro Kjeldahl digestion and distillation system. The available phosphorus was measured by the colorimetric molybdate blue method [33]. The exchangeable bases, namely, potassium (K), calcium (Ca), and magnesium (Mg), were extracted from the organic amendments using neutral normal ammonium acetate [34] and measured by titration with Trilon [35].

### 2.7. Statistical Data Analysis

The experiment was subjected to analysis of variance in a complete randomized block design and data were analyzed using the R-program (version 4.1.3, 2022). To determine the significant differences between treatment means, Fisher's range test at a 5% significance level (*P* < 0.05) was applied. Correlation analysis was also computed to see the relationship between the principal components.

## 3. Results and Discussion

### 3.1. Organic Amendment Properties

**Figure 2:**
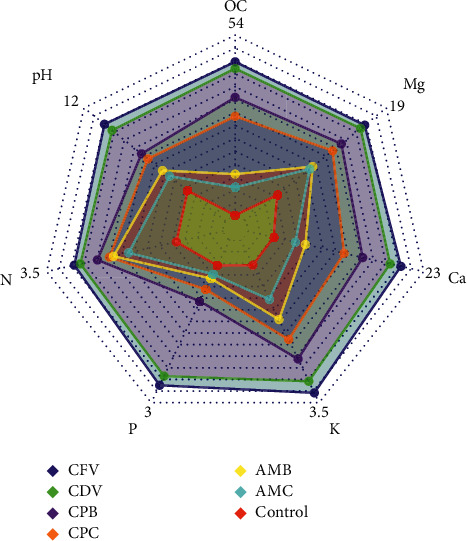
Radar plot for soil properties of different organic amendments.

There were highly significant (*P* < 0.001) differences among the soil properties of the organic amendments. The highest percentage of organic carbon was registered in the coffee pulp fetida vermicompost (47.51 ± 0.06) followed by coffee pulp *Dendrobaena veneta* vermicompost (45.86 ± 0.28), while the lowest values were recorded in the control treatment (10.07 ± 0.19) and animal manure compost (16.90 ± 0.15). Besides, the maximum percentage of total nitrogen content (2.95 ± 0.07), percentage of available phosphorus (2.68 ± 0.06), and the exchangeable potassium (3.29 ± 0.04 cmol_(+)_/kg of soil), calcium (20.68 ± 1.01 cmol_(+)_/kg), and magnesium (17.01 ± 0.15 cmol_(+)_/kg) were found in coffee pulp fetida vermicompost whereas the mentioned soil chemical properties were statistically lower in the control treatment (topsoil). In addition, the higher pH values were measured in coffee pulp fetida vermicompost (10.84 ± 0.05) and coffee pulp *Dendrobaena veneta* vermicompost (10.41 ± 0.04), while the lower pH values were found in the topsoil (6.12 ± 0.04) and animal manure compost (7.15 ± 0.07) organic amendments (Figure 2).

### 3.2. Plant Height (cm)

**Figure 3:**
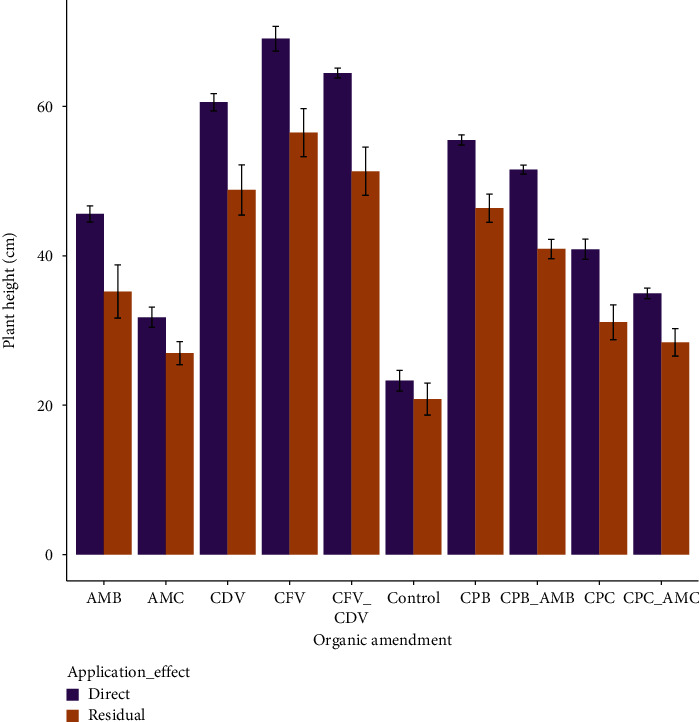
Response of hot pepper plant height to direct application and residual effects of organic amendments.

The interactive effect of growth media and application effect on mean plant height showed highly significant difference (*P* < 0.001). The tallest plant height (69.07 ± 1.65 cm) was recorded in the direct application of coffee pulp fetida vermicompost followed by in the combination of coffee pulp vermicompost using fetida and *Dendrobaena veneta* earthworms applied directly (64.47 ± 0.65 cm) while the shortest plant height was registered in the control treatments of residual (20.82 ± 2.14 cm) and direct (23.27 ± 1.38 cm) effects. Likewise, plant height in the directly applied coffee pulp fetida vermicompost was higher by 69.86% than plant height in the residual control treatment (Figure 3).

Plant height was significantly influenced by the earthworm types in coffee pulp composts. Longer plants were observed in coffee pulp compost produced by fetida (69.07 ± 1.65) than *Dendrobaena veneta* (48.82 ± 3.37) earthworms in the direct and residual effects, respectively. Coffee pulp and animal manure in biochar forms were also statistically different from the same growth media in the form of compost. Direct application of animal manure biochar had a higher plant height (45.60 ± 1.08) than the residual effect of animal manure in the form of compost (26.98 ± 1.54). In addition, plant height in the combination of coffee pulp biochar and animal manure biochar applied directly (51.53 ± 0.60) was higher by 44.91% than plant height in the residual effect of combined coffee pulp compost and animal manure compost (28.39 ± 1.84) (Table 1).

Generally, the longest plant heights were observed in the organic amendments consisting of vermicompost followed by biochar, whereas the shortest heights were found in the control treatment and in the conventional composts during direct application and residual effects, possibly due to the significant variation in organic carbon and total nitrogen content, which led to increasing plant height by improving soil fertility and nutrient uptake capacity [36]. The alkaline nature of vermicompost is accountable to produce more root exudates and the multiplication of soil microbes [37]. The availability of exchangeable calcium, magnesium, and potassium in the organic amendments may neutralize organic acids and improve the absorption of other nutrients [38].

The result of this study was also in line with several research findings [39–47], which conveyed that the application of organic fertilizer, particularly vermicompost, improved plant heights. Similar findings were also explored by Maru et al. [48]; Sikder and Joardar [49]; and Bhattarai et al. [50], who determined that the application of organic fertilizer in biochar form increased plant height in pea and rice crops. This might be due to the availability of high organic matter and total nitrogen content [36], which enhance the capacity to easily uptake nutrients [51, 52], maintain soil moisture, and improve fertility [53] that eventually advances growth media porosity, aeration, and water retention capacity [54]. Organic amendments provide growth stimulants for plants to grow and resist unfavorable conditions as supported by Pampuro et al. [55]. It also helps maintain the soil's biological balance, as well as prevents the growth of harmful microorganisms in the soil through destruction and antagonistic and inhibiting mechanisms [53]. Besides, the differences in plant height could possibly be due to the nature of the biochar soil amendment which improves soil nutrients and increased plant growth parameters as supported by Steiner et al. [56]; Abiven et al. [57]; and Agegnehu et al. [58]. Accordingly, Chemura et al. [59] reported that the excessive use of organic amendments may result in the accumulation of more vegetative growth than the increase in crop yield. Generally, Singh et al. [60] concluded that both soil and agronomic sustainability can be maintained by using a combination of organic fertilization with reduced inputs from mineral fertilizers.

### 3.3. Number of Leaves per Plant

**Figure 4:**
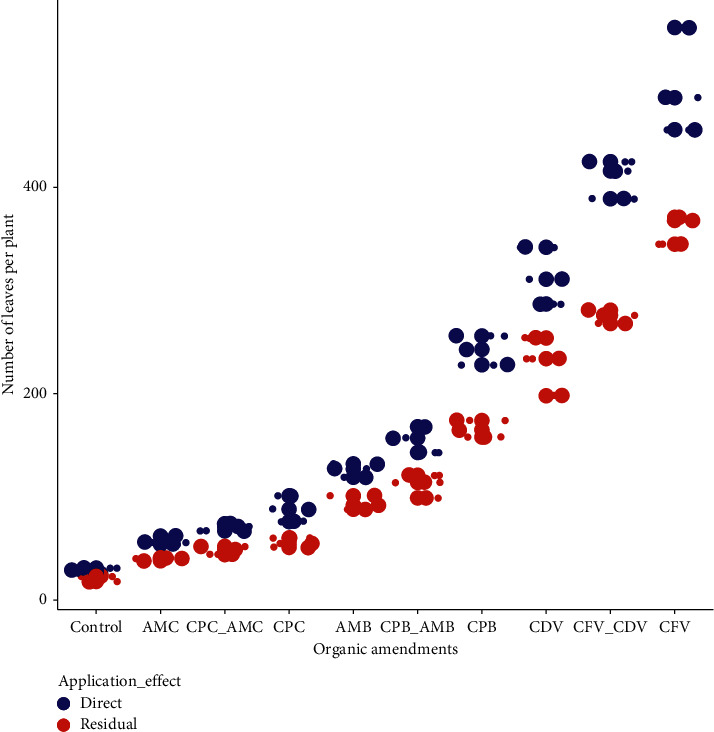
The influence of organic amendments on hot pepper leaf number.

The combined influence of organic amendment and application effect on the mean number of leaves has shown a significant difference (*P* < 0.05). The maximum number of leaves (499.29 ± 50.64) was observed in the direct application of coffee pulp fetida vermicompost followed by the combination of coffee pulp vermicompost using fetida and *Dendrobaena veneta* earthworms applied directly (410.02 ± 18.73) while the minimum leaf number was scored in the control treatments of residual (20.03 ± 2.65) and direct (28.64 ± 2.52) effects followed by the residual effect (39.67 ± 1.53) and direct application (57.32 ± 4.16) of animal manure compost. The number of leaves in directly applied coffee pulp fetida vermicompost was higher by 95.99% than the leaf number in the residual control treatment (Figure 4).

In a general context, the higher number of leaves was counted in the organic amendments with vermicompost followed by biochar whereas the lowest number of leaves were observed in the control treatment and in the conventional coffee husk and animal manure composts both in the direct application and residual effects, probably due to variation in organic carbon, total nitrogen content, exchangeable potassium, calcium, magnesium, and alkaline nature of the pH (Figure 2). Leaf number was also statistically influenced by the earthworm types in coffee pulp composts. In this sense, the higher number of leaves was observed in coffee pulp compost produced by fetida (499.29 ± 50.64) than *Dendrobaena veneta* (228.69 ± 28.38) earthworms in the direct and residual effects, respectively.

The number of leaves in coffee pulp and animal manure in biochar forms also varied from the same growth media when applied in the form of compost. Direct application of animal manure biochar had a higher leaf number (126.00 ± 6.56) than the residual effect of animal manure compost (39.67 ± 1.53). In addition, leaves in the combination of coffee pulp biochar and animal manure biochar applied directly (156.08 ± 12.53) were higher by 69.04% than leaf number in the residual effect of combined coffee pulp and animal manure (48.33 ± 4.04) composts. However, leaf number in animal manure compost was not a significant difference compared to coffee pulp compost during the two growing seasons (Table 1).

This finding is in agreement with that of Amiri et al. [61], who reported that direct application of organic fertilizer of vermicompost increases leaf numbers of cabbage. In addition, Hameeda et al. [62]; Ansari and Jaikishun [63]; Fritz et al. [64]; and Govindapillai et al. [65] stated that soil amended with vermicompost increases the number of leaves per plant compared with soil without organic fertilizer application. A similar result was also reported by Prasad et al. [66], who depicted that the combined application of biochar and vermicompost increases the number of leaves. This could be because of the availability of organic matter in vermicompost and biochar and their capacity to easily uptake nutrients and maintain soil moisture that eventually increases the number of leaves per plant. Vennila et al. [67] also confirmed that the presence of nutrients, hormones, and biochemical content in vermicompost promotes better plant growth and nutrient absorption. This might also be due to the solar brightness and photosynthetically active radiation [68, 69] as the leaf is responsible for carbon assimilation, nutrient and water use, evapotranspiration, development rate, and photosynthetic efficiency [70].

### 3.4. Number of Branches per Plant

**Figure 5:**
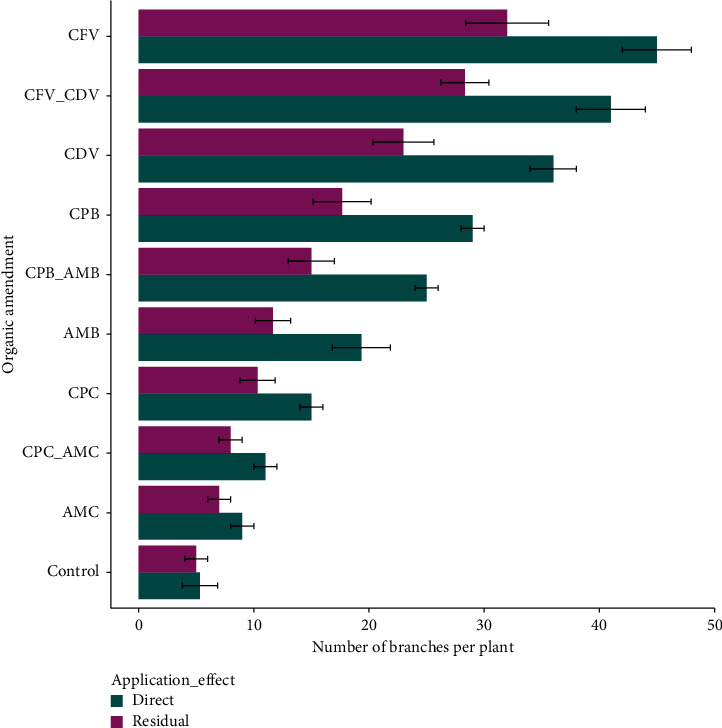
Number of branches per plant in different organic amendments.

The interaction effect of organic amendment and application effect on the mean number of branches per plant were highly significant (*P* < 0.001) variation (Table 1). The higher number of branches (45.02 ± 3.04) was recorded in the direct application of coffee pulp fetida vermicompost followed by the combination of coffee pulp vermicompost using fetida and *Dendrobaena veneta* earthworms applied directly (41.06 ± 3.11) and coffee pulp *Dendrobaena veneta* vermicompost (35.96 ± 1.97), while the lower branch number was found in the control treatments of residual (5.04 ± 0.96) and direct (5.32 ± 1.53) effects followed by the residual effect (7.05 ± 0.93) and direct application (9.04 ± 1.01) of animal manure composts. The number of branches per plant in directly applied coffee pulp fetida vermicompost was higher by 88.80% than the leaf number in the residual control treatment (Figure 5).

Generally, many branches were counted in the organic amendments consist vermicompost and biochar whereas few numbers of branches were observed in the control treatment and conventional coffee husk and animal manure composts during direct application and in the residual effects. This could be possibly due to organic matter variation among the growth media (Figure 2). The earthworm types had a significant influence on branching the plant. Likewise, direct application of coffee pulp compost produced by fetida (45.02 ± 3.04) showed a higher number of branches 28.92% than using *Dendrobaena veneta* (32.00 ± 3.61) earthworms in the residual effect. The number of branches in coffee pulp and animal manure in biochar forms also varied from the same growth media when applied in the form of compost. In addition, a significantly higher number of branches were found in the direct application of animal manure biochar (19.30 ± 2.52) than in the residual effect of animal manure compost (7.05 ± 0.93). Besides, branches in the combination of coffee pulp biochar and animal manure biochar applied directly (24.98 ± 1.10) was notably higher than branch number in the residual effect of combined coffee pulp and animal manure (8.02 ± 1.00) composts (Table 1).

This finding is in accordance with Shadanpour et al. [71], who reported that direct application of organic fertilizer chiefly vermicompost increased the number of branches per plant of okra and chili pepper. Govindapillai et al. [65] also stated that the number of branches was increased in soils amended by organic fertilizer than the control treatment. Comparatively similar results were obtained by Singh et al. [72], who determined that the application of organic amendments contributes to the availability of nutrients and microbial population to increase the number of branches per plant in chili pepper. Another research finding by Zheng et al. [73] and Chan et al. [74] showed that the direct application of organic fertilizer of biochar produced from plants and animals has a positive effect on plant growth parameters than the control. The increment in a number of branches per plant might be possibly due to the availability of plant nutrients released by microorganisms.

### 3.5. Days to 50% Flowering

**Figure 6:**
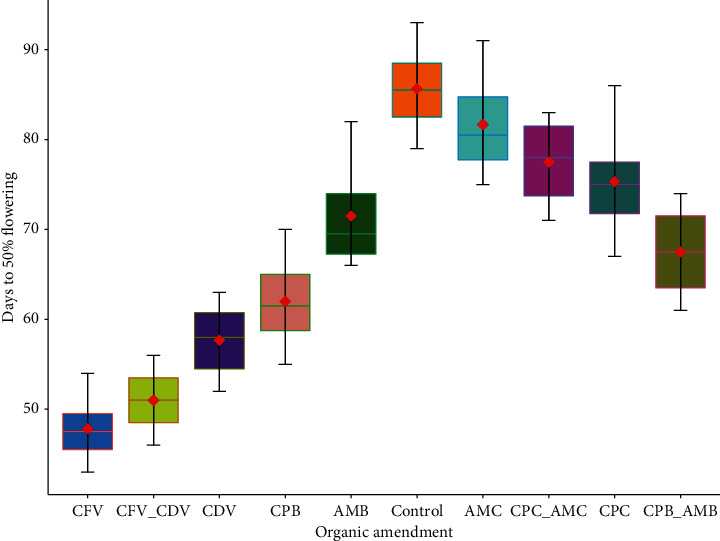
Days to 50% flowering in different organic amendments.

The effect of organic amendments in the days to 50% flowering has shown a highly significant difference (*P* < 0.001). The earliest days to 50% flowering were recorded in coffee pulp fetida vermicompost (47.83 ± 3.87) and a combination of coffee pulp fetida and *Dendrobaena veneta* vermicompost (51.00 ± 3.74) while the later days to 50% flowering were observed in the control treatment (85.68 ± 5.05) and animal manure compost (81.67 ± 5.92). Coffee pulp fetida vermicompost applied in hot pepper flowered earlier by 44.18% than hot pepper is grown using topsoil solely (Figure 6).

The days to 50% flowering in the combination of coffee pulp biochar and animal manure biochar (67.50 ± 5.24) was earlier than in the combination of coffee pulp compost and animal manure compost (77.52 ± 4.93). In the same way, there was a significant difference between animal manure biochar and animal manure compost in days to 50% flowering. Days to 50% flowering in animal manure biochar (71.48 ± 6.09) were statistically higher than in animal manure compost (81.65 ± 5.92). On top of that, significantly higher days to 50% flowering were found in coffee pulp biochar (62.00 ± 5.40) than in coffee pulp compost (75.33 ± 6.50). The days to 50% flowering were also significantly influenced by the earthworm types in coffee pulp growth media. Earlier days to 50% flowering were observed in coffee pulp vermicompost produced by fetida (47.83 ± 3.87) than *Dendrobaena veneta* (57.67 ± 4.32) earthworms (Figure 6).

This result is in agreement with the result of Taleb [75], who reported that longer days to 50% flowering were observed using conventional fertilizers while the days become short when utilizing poultry manure organic fertilizer. A rapid and significantly different (26 days) first flowering has been reported by using vermicompost than other organic fertilizers [76]. A similar finding has been reported by Kiros et al. [77], who suggested that vermicompost significantly shorten the length of days to 50% flowering. The decline in the number of days to 50% flowering when utilizing organic amendment as a growth media might be due to continued decomposition of the growth media application resulting in increasing soil temperature, which is important to increase the amount of potassium in the rhizosphere and fasten the blooming [75].

**Figure 7:**
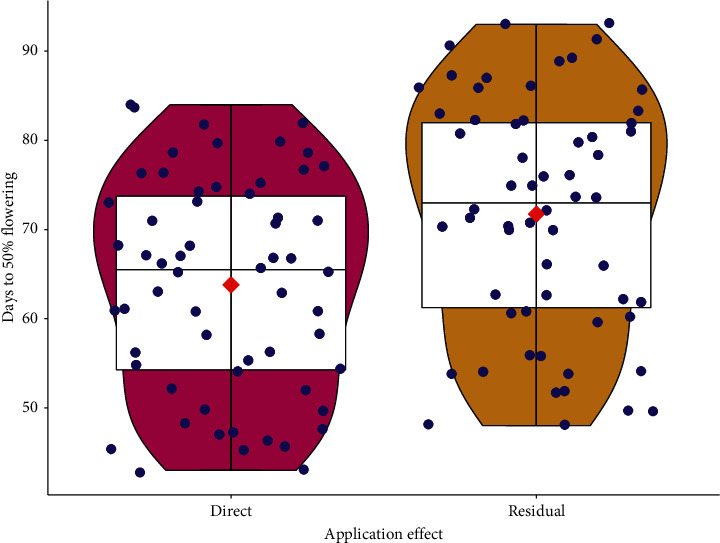
Response of days to 50% flowering to organic amendment application effects, combined graphs of boxplot, geom violin, and geom point; red diamond is the mean values.

The influence of the application effect in mean days to 50% flowering was highly significant (*P* < 0.001). All organic amendments applied directly had notably earlier days to 50% flowering than the residual effects of the organic amendments. Apparently, a day to 50% flowering in the direct application of organic amendments (63.80 ± 12.13) was higher by 10.06% than the residual (71.73 ± 13.25) effects (Figure 7).

### 3.6. Total Fresh Biomass Weight (g)

**Figure 8:**
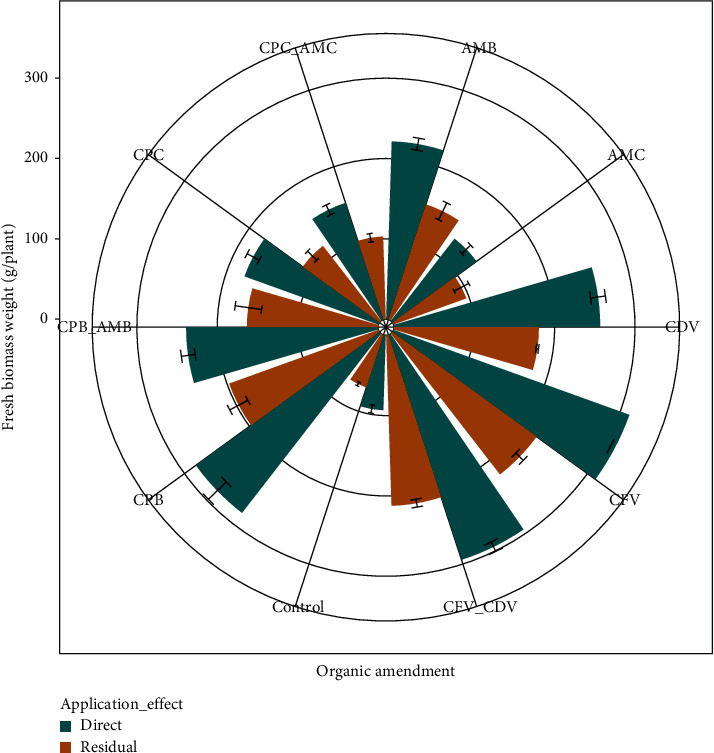
Response of fresh biomass weight to direct and residual effects of organic amendments.

The interaction effect of organic amendment and application effect on mean fresh biomass weight per plant showed highly significant (*P* < 0.001) difference. The heaviest fresh biomass weight (312.00 ± 5.57) was measured in the direct application of coffee pulp fetida vermicompost followed by in the combination of coffee pulp vermicompost using fetida and *Dendrobaena veneta* earthworms applied directly (294.32 ± 6.50) while the lightest biomass weight was registered in the control treatments of residual (68.67 ± 2.52) and direct (93.34 ± 5.13) effects. Likewise, fresh biomass weight in the directly applied coffee pulp fetida vermicompost was higher by 77.99% than in the residual control treatment (Figure 8).

Fresh biomass weight was significantly influenced by the earthworm types in coffee pulp composts. Heavier plants were observed in coffee pulp compost produced by fetida (312.00 ± 5.57) than *Dendrobaena veneta* (180.33 ± 1.53) earthworms in the direct and residual effects, respectively. Direct application of animal manure biochar had a higher biomass weight (221.30 ± 7.51) than the residual effect of animal manure in the form of compost (96.29 ± 10.21). In addition, biomass weight in the combination of coffee pulp biochar and animal manure biochar applied directly (238.68 ± 8.62) was higher by 56.85% than biomass weight in the residual effect of combined coffee pulp compost and animal manure (103.00 ± 5.29) compost (Figure 8).

Generally, higher biomass weights were found in the organic amendments with vermicompost and biochar whereas lower values were weighed in the control treatment and conventional composts during direct application and residual effects, possibly due to the significant variation in growth media physicochemical properties, such as organic carbon, total nitrogen content, and bulk density which led to increasing fresh biomass weight by improving soil fertility and nutrient uptake capacity [36].

This result was in agreement with several research findings [7, 76–80], who concisely reported that there is an increment in total plant fresh biomass weight when organic fertilizer specially vermicompost or biochar is applied. The advancing trend of pepper total biomass was mainly for the account of the increased mass of the above and underground organs [82]. Besides, Alviana et al. [80] reported that the fresh biomass weight of plants with fertilizer treatment up to the highest dosage increased the hot pepper plant weight linearly.

Not only in hot pepper, the effects of organic fertilizer significantly influenced the biomass yield of haricot bean as well [83]. The authors reported that biomass production has shown an increasing trend with an increasing rate of organic fertilizer in haricot bean. They succinctly concluded that the biomass weight had a significant and positive association with the plant height, leaf area index, branch number, root length, and other yield attributes. Yang et al. [84] further substantiated that the higher leaf area index of plants enabled plants to intercept more of the available radiation for the production of total plant biomass. The current result in total fresh biomass weight could be due to myriad contributions, chiefly the increases in nutrient absorption and mineral uptake that results from the direct interaction between organic soil nutrient and plant growth as supported by Liu et al. [79]. Accordingly, Khaitov et al. [78] concluded that the excessive use of organic manure may result in the accumulation of more vegetative biomass than the increase in hot pepper yield value.

### 3.7. Number of Fruits per Plant

**Figure 9:**
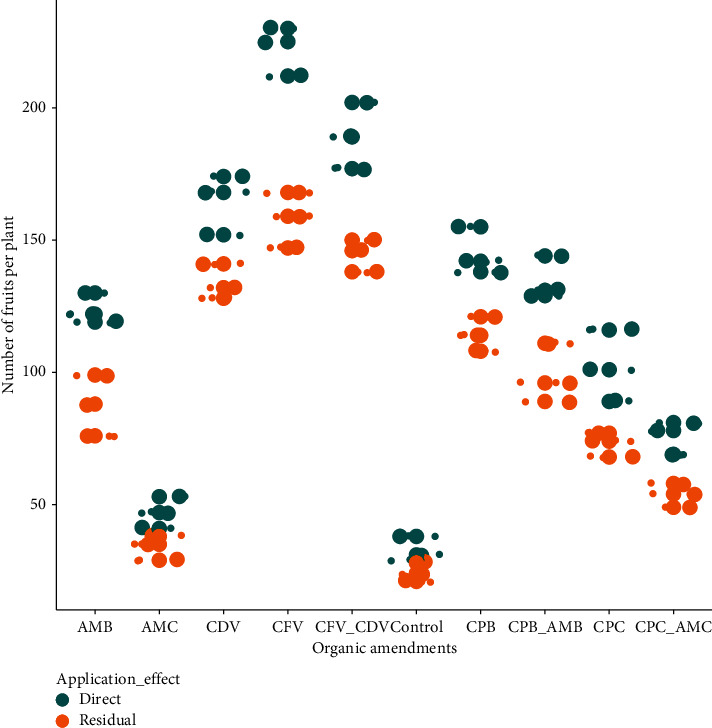
Number of fruits per plant in direct and residual effects of organic amendments.

The combined influence of organic amendment and application effect on the mean number of fruits per plant showed a significant (*P* < 0.001) variation. The maximum number of fruits (222.31 ± 9.29) was recorded in the direct application of coffee pulp fetida vermicompost followed by combined coffee pulp vermicompost using fetida and *Dendrobaena veneta* earthworms applied directly (189.28 ± 12.49) and direct application of coffee pulp dendrovenbabenta vermicompost (164.68 ± 11.38) while the minimum fruit number was scored in the control treatments of residual (24.30 ± 3.51) and direct (32.67 ± 4.73) effects followed by the residual effect (34.07 ± 4.58) and direct application (47.00 ± 6.00) of animal manure compost. The number of fruits in directly applied coffee pulp fetida vermicompost was higher by 89.07% than the fruit number in the residual control treatment (Figure 9).

The higher number of fruits was counted in the organic amendments with vermicompost followed by biochar whereas the lower number of fruits was observed in the control treatment and the conventional coffee husk and animal manure composts both in the direct application and residual effects. Fruit number was also statistically influenced by the earthworm types in coffee pulp composts. In this sense, higher number of fruits was observed in coffee pulp compost produced by fetida (222.31 ± 9.29) than *Dendrobaena veneta* (133.67 ± 6.65) earthworms in the direct and residual effects, respectively (Table 1). Direct application of animal manure biochar had a higher fruit number (123.68 ± 5.69) than the residual effect of animal manure compost (34.07 ± 4.58). In addition, fruits in the combination of coffee pulp biochar and animal manure biochar applied directly (134.70 ± 8.14) were higher by 60.14% than the fruits number in the residual effect of combined coffee pulp and animal manure (53.69 ± 4.50) composts (Figure 9).

This result is in coherence with Adhikari et al. [76], who explored that the maximum number of hot pepper fruits was observed using vermicompost than other growth media compositions. Nawrin et al. [85] also reported that higher fruits per plant were recorded by applying 0.5 kg per hectare boron and 5 ton per hectare vermicompost. Mkhabela et al. [43] found a similar result when they looked at how much more fruit was produced using vermicompost compared to other organic amendments.

### 3.8. Single Fruit Length (cm)

**Figure 10:**
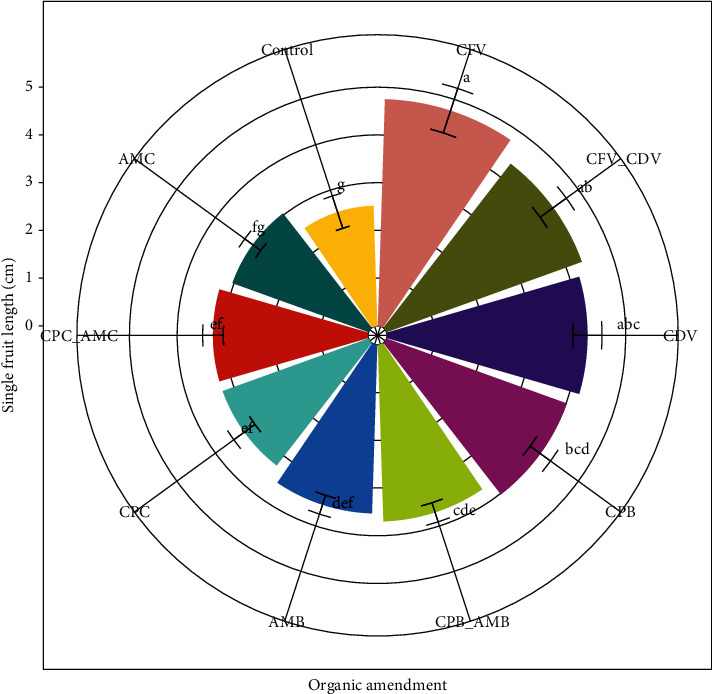
Response of hot pepper single fruit length to different organic amendments.

The effect of organic amendment in the single fruit length has shown a highly significant difference (*P* < 0.001). The maximum values were observed in the coffee pulp fetida vermicompost (4.75 ± 0.49) followed by in the combination of coffee pulp fetida vermicompost and coffee pulp *Dendrobaena veneta* vermicompost (4.35 ± 0.34) while the minimum values were recorded in the control treatment (2.52 ± 0.35) and animal manure compost (3.03 ± 0.21). The single fruit length in the coffee pulp fetida vermicompost was higher by 46.95% than the fruit length in the control treatment (Figure 10).

Single fruit length in the combination of coffee pulp biochar and animal manure biochar (3.70 ± 0.21) was higher than in the combination of coffee pulp compost and animal manure compost (3.25 ± 0.22). Likewise, the single fruit length in animal manure biochar (3.53 ± 0.21) was higher than applying animal manure in the form of compost (3.03 ± 0.21). In addition, significantly higher single fruit length was found in coffee pulp biochar (4.02 ± 0.26) than in coffee pulp compost (3.25 ± 0.27). The single fruit length was also significantly influenced by the earthworm types in coffee pulp growth media. Significantly higher single fruit length was observed in coffee pulp vermicompost produced by fetida (4.75 ± 0.49) than *Dendrobaena veneta* (4.20 ± 0.30) earthworms (Figure 10).

The present result is in line with Murphy [44], who reported that the availability of essential nutrients with enough hormones found in organic fertilizer of vermicompost could improve vegetative and reproductive growth, particularly fruit length of vegetable crops. Other studies have also shown that organic fertilizer application increases fruit length and other plant growth parameters. A similar result was obtained by Hazrat et al. [86], who indicated that the release of essential macronutrients, such as nitrogen, phosphorous, and potassium, in organic amendments increased in fruit length of crops.

**Figure 11:**
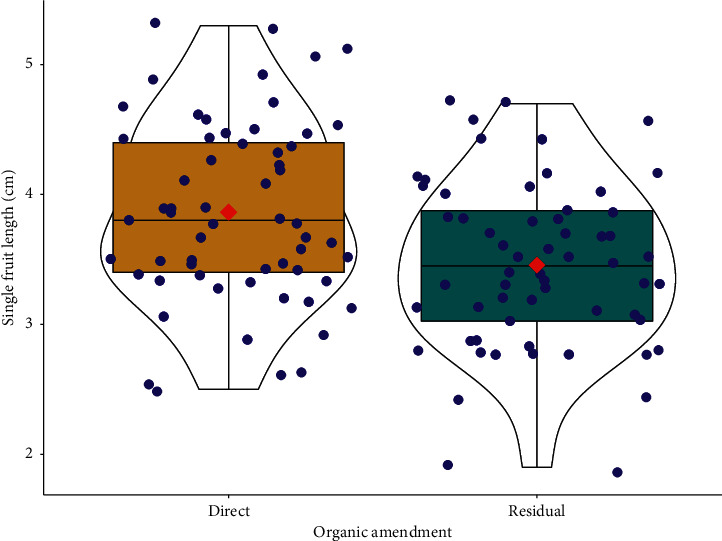
Response of hot pepper single fruit length to organic amendment application effects.

The application effect on mean single fruit length was highly significant (*P* < 0.001). All organic amendments applied directly had notably longer fruits than the residual effects of the organic amendments. In other words, single fruit length in the direct application of organic amendments (3.86 ± 0.71) was higher by 10.62% than the residual (3.45 ± 0.65) effects (Figure 11).

### 3.9. Fruit Yield (Ton/Hectare)

The interactive effect of organic amendment and application effect on mean yield per hectare showed highly significant (*P* < 0.001) difference. The maximum fruit yield per hectare (26.21 ± 1.20) was registered in the direct application of coffee pulp fetida vermicompost followed by the combination of coffee pulp vermicompost using fetida and *Dendrobaena veneta* earthworms applied directly (21.56 ± 2.57) while the minimum hot pepper fruit yield per hectare was recorded in the control treatments of residual (0.56 ± 0.07) and direct (0.70 ± 0.13) effects. Directly applied coffee pulp fetida vermicompost had a higher yield of 97.86% than fruit yield in the residual control treatment (Table 1).

Fruit yield was significantly influenced by the earthworm types in coffee pulp composts. Higher yields were found in coffee pulp compost produced by fetida (26.21 ± 1.20) than *Dendrobaena veneta* (12.37 ± 0.59) earthworms in the direct and residual effects, respectively. Besides, the direct application of animal manure biochar had a higher yield (6.053 ± 0.28) than the residual effect of animal manure in the form of compost (1.00 ± 0.12). In addition, fruit yield per hectare in the combination of coffee pulp biochar and animal manure biochar applied directly (7.68 ± 0.35) was higher by 75.65% than the yield in the residual effect of combined coffee pulp compost and animal manure (1.87 ± 0.15) compost (Table 1).

Interestingly, the highest fruit yields were observed in the organic amendments consisting of vermicompost followed by biochar whereas the lowest yields were found in the control treatment and in the conventional composts during direct application and residual effects, possibly due to the significant variation in growth media soil nutrition status and nutrient uptake capacity [36]. The alkaline nature of vermicompost is accountable to produce more root exudates and the multiplication of soil microbes [37]. The availability of exchangeable calcium, magnesium, and potassium in the organic amendments may neutralize organic acids and improve the absorption of other nutrients [38].

The finding of this study is in accordance with several research findings that concisely reported that direct application of organic fertilizer particularly vermicompost and biochar and their residual effects increased the marketable yield of okra [87], strawberry [72], eggplant [88], potato [89], cucumber [90], *Abelmoschus esculentus* [47], and lettuce [91]. Uma and Malathi [92] found that plants of *Amaranthus* sp. in plots receiving vermicompost had higher yields as compared to plants in plots receiving chemical fertilizers. According to Adhikari et al. [76], sweet pepper plants treated with organic fertilizers showed greater yield values than plants treated with chemical fertilizer. Similar results were reported by Azarmi et al. [90], who discovered that tomato vegetative growth and productivity were advanced by vermicompost treatment directly as well as by its residual influence.

Generally, organic amendments particularly the vermicompost played a vital role in increasing hot pepper fruit yield per hectare both in the first season of direct application and grown using the residual effects of the previously applied organic amendments, probably due to higher soil nutritional value in vermicompost than traditional composts, high porosity, aeration, drainage, and water-holding capacity, presence of microbiota particularly fungi, bacteria, and actinomycetes, availability of nutrients, such as nitrates, phosphates, and exchangeable calcium and soluble potassium, and the presence of plant growth regulators [93–97].

## 4. Conclusion

Coffee pulp and animal manure wastes produced from several processing industries were the main environmental challenges in the Gedeo zone. On the other hand, these wastes are vital if there is proper management and use as an organic fertilizer to replace the expensive, easily inaccessible, and environmentally unfriendly chemical fertilizers. Using earthworms to produce vermicompost is also a new innovative technology in smart agriculture for sustainable production. In this study, we found hot pepper early maturation, higher growth rate, and a huge amount of yield by direct application of vermicompost particularly produced by fetida earthworm. The coffee pulp and animal manure wastes used in the form of biochar were also promising for hot pepper vegetative and reproductive growth. Likewise, soil fertility and agronomic sustainability can be maintained by combined application of organic amendment. The residual effects of the organic amendments played a vital role to produce a higher amount of hot pepper yield and yield components compared to the topsoil. Thus, recycling wastes or converting them into reusable organic fertilizers could be a master key to opening triple doors of a clean environment, healthy food, and high production.

## Figures and Tables

**Table 1:** Interaction effect of organic amendment and application effect on hot pepper plant height, leaf number, branch number, biomass weight, fruit number, and yield.

Organic amendment	Application effect	Plant height (cm)	Leaf number	Branch number	Biomass weight (g)	Fruit number	Yield (ton/ha)
AMB	Direct	45.60 ± 1.08^f^	126.00 ± 6.56^h^	19.30 ± 2.52^g^	221.30 ± 7.51^e^	123.68 ± 5.69^fg^	6.053 ± 0.28^g^
AMC	Direct	31.77 ± 1.36^ij^	57.32 ± 4.16^kl^	9.04 ± 1.01^ijk^	129.31 ± 7.50^j^	47.00 ± 6.00^mn^	1.31 ± 0.06^lm^
CDV	Direct	60.57 ± 1.15^c^	313.33 ± 27.57^d^	35.96 ± 1.97^c^	256.67 ± 9.29^c^	164.68 ± 11.37^c^	16.67 ± 1.19^c^
CFV	Direct	69.07 ± 1.65^a^	499.29 ± 50.64^a^	45.02 ± 3.04^a^	312.00 ± 5.57^a^	222.31 ± 9.29^a^	26.21 ± 1.20^a^
CPB	Direct	55.50 ± 0.70^d^	242.33 ± 14.01^f^	29.00 ± 1.03^de^	282.33 ± 15.50^b^	144.79 ± 8.89^de^	11.31 ± 0.73^e^
CPC	Direct	40.87 ± 1.36^g^	88.30 ± 12.50^ij^	15.05 ± 1.07^h^	177.00 ± 8.54^gh^	102.00 ± 13.53^hi^	4.08 ± 0.11^ij^
CFV_CDV	Direct	64.47 ± 0.65^b^	410.02 ± 18.73^b^	41.06 ± 3.11^b^	294.32 ± 6.50^b^	189.28 ± 12.49^b^	21.56 ± 2.57^b^
CPB_AMB	Direct	51.53 ± 0.60^e^	156.08 ± 12.53^g^	24.98 ± 1.10^f^	238.68 ± 8.62^d^	134.70 ± 8.14^ef^	7.68 ± 0.35^f^
CPC_AMC	Direct	34.97 ± 0.70^hi^	70.65 ± 3.51^jk^	10.89 ± 0.91^ij^	152.67 ± 6.66^i^	75.88 ± 6.24^kl^	2.49 ± 0.41^kl^
Control	Direct	23.27 ± 1.38^m^	28.64 ± 2.52^mn^	5.32 ± 1.53^l^	93.34 ± 5.13^k^	32.67 ± 4.73^o^	0.70 ± 0.13^m^
AMB	Residual	35.21 ± 3.56^h^	93.68 ± 6.65^ij^	11.68 ± 1.53^i^	150.66 ± 11.68^i^	87.66 ± 11.50^jk^	4.48 ± 0.57^hi^
AMC	Residual	26.98 ± 1.54^l^	39.67 ± 1.53^lmn^	7.05 ± 0.93^kl^	96.29 ± 10.21^k^	34.07 ± 4.58^no^	1.00 ± 0.12^m^
CDV	Residual	48.82 ± 3.37^ef^	228.69 ± 28.38^f^	22.88 ± 2.65^f^	180.33 ± 1.53^g^	133.67 ± 6.65^ef^	12.37 ± 0.59^e^
CFV	Residual	56.48 ± 3.21^d^	361.28 ± 14.22^c^	32.00 ± 3.61^d^	221.67 ± 6.51^e^	157.98 ± 10.54^cd^	15.59 ± 0.68^cd^
CPB	Residual	46.37 ± 1.89^f^	165.67 ± 8.02^g^	17.66 ± 2.52^gh^	197.29 ± 12.74^f^	114.28 + 6.51^gh^	7.39 ± 0.60^f^
CPC	Residual	31.11 ± 2.33^jk^	55.36 ± 4.51^klm^	10.30 ± 1.50^ij^	118.02 ± 7.94^j^	73.03 ± 4.58^l^	3.00 ± 0.30^jk^
CFV_CDV	Residual	51.31 ± 3.22^e^	274.97 ± 6.56^e^	28.33 ± 2.08^e^	212.28 ± 4.93^e^	144.65 ± 6.11^de^	14.44 ± 0.88^d^
CPB_AMB	Residual	40.91 ± 1.28^g^	111.27 ± 11.24^hi^	15.00 ± 1.99^h^	163.00 ± 16.64^hi^	98.68 ± 11.24^ij^	5.57 ± 0.28^gh^
CPC_AMC	Residual	28.39 ± 1.84^kl^	48.33 ± 4.04^klm^	8.02 ± 1.00^jkl^	103.00 ± 5.29^k^	53.69 ± 4.50^m^	1.87 ± 0.15^klm^
Control	Residual	20.82 ± 2.14^m^	20.03 ± 2.65^n^	5.04 ± 0.96^l^	68.67 ± 2.52^l^	24.30 ± 3.51^o^	0.56 ± 0.07^m^
Mean		43.20	169.55	19.68	183.45	107.97	8.22
CV		4.58	9.87	10.03	4.84	7.74	9.77
LSD		3.26	27.60	3.26	14.65	13.80	1.33
*P*-value		^*∗∗∗*^	^*∗∗∗*^	^*∗∗∗*^	^*∗∗∗*^	^*∗∗∗*^	^*∗∗∗*^

Means within a column followed by the same letter(s) are not significantly different at the 5% LSD test.

## Data Availability

The data that support the findings of this study are available upon request from the corresponding author.
